# Applications of Non-invasive Neuromodulation for the Management of Disorders Related to COVID-19

**DOI:** 10.3389/fneur.2020.573718

**Published:** 2020-11-25

**Authors:** Abrahão Fontes Baptista, Adriana Baltar, Alexandre Hideki Okano, Alexandre Moreira, Ana Carolina Pinheiro Campos, Ana Mércia Fernandes, André Russowsky Brunoni, Bashar W. Badran, Clarice Tanaka, Daniel Ciampi de Andrade, Daniel Gomes da Silva Machado, Edgard Morya, Eduardo Trujillo, Jaiti K. Swami, Joan A. Camprodon, Katia Monte-Silva, Katia Nunes Sá, Isadora Nunes, Juliana Barbosa Goulardins, Marom Bikson, Pedro Sudbrack-Oliveira, Priscila de Carvalho, Rafael Jardim Duarte-Moreira, Rosana Lima Pagano, Samuel Katsuyuki Shinjo, Yossi Zana

**Affiliations:** ^1^Center for Mathematics, Computation and Cognition, Federal University of ABC, São Bernardo do Campo, Brazil; ^2^NAPeN Network (Rede de Núcleos de Assistência e Pesquisa em Neuromodulação), Brazil; ^3^Brazilian Institute of Neuroscience and Neurotechnology Centros de Pesquisa, Investigação e Difusão - Fundação de Amparo à Pesquisa do Estado de São Paulo (BRAINN/CEPID-FAPESP), University of Campinas, Campinas, Brazil; ^4^Laboratory of Medical Investigations 54 (LIM-54), São Paulo University, São Paulo, Brazil; ^5^Specialized Neuromodulation Center—Neuromod, Recife, Brazil; ^6^Graduate Program in Physical Education, State University of Londrina, Londrina, Brazil; ^7^School of Physical Education and Sport, University of São Paulo, São Paulo, Brazil; ^8^Laboratory of Neuroscience, Hospital Sirio-Libanes, São Paulo, Brazil; ^9^Centro de Dor, LIM-62, Departamento de Neurologia, Faculdade de Medicina FMUSP, Universidade de São Paulo, São Paulo, Brazil; ^10^Serviço Interdisciplinar de Neuromodulação, Laboratório de Neurociências (LIM-27), Instituto Nacional de Biomarcadores em Neuropsiquiatria, São Paulo, Brazil; ^11^Instituto de Psiquiatria, Hospital das Clínicas HCFMUSP, Faculdade de Medicina, Universidade de São Paulo, São Paulo, Brazil; ^12^Department of Psychiatry, Medical University of South Carolina, Charleston, SC, United States; ^13^Instituto Central, Hospital das Clínicas HCFMUSP, Faculdade de Medicina, Universidade de São Paulo, São Paulo, Brazil; ^14^Graduate Program in Collective Health, Federal University of Rio Grande do Norte, Natal, Brazil; ^15^Edmond and Lily Safra International Neuroscience Institute, Santos Dumont Institute, Macaiba, Brazil; ^16^Department of Biomedical Engineering, The City College of New York of CUNY, New York, NY, United States; ^17^Laboratory for Neuropsychiatry and Neuromodulation, Massachusetts General Hospital and Harvard Medical School, Boston, MA, United States; ^18^Applied Neuroscience Laboratory, Universidade Federal de Pernambuco, Recife, Brazil; ^19^Escola Bahiana de Medicina e Saúde Pública, Salvador, Brazil; ^20^Department of Physiotherapy, Pontifícia Universidade Católica de Minas Gerais, Betim, Brazil; ^21^Universidade Cruzeiro do Sul (UNICSUL), São Paulo, Brazil; ^22^Faculdade de Medicina FMUSP, Universidade de São Paulo, São Paulo, Brazil; ^23^Division of Rheumatology, Faculdade de Medicina FMUSP, Universidade de São Paulo, São Paulo, Brazil

**Keywords:** coronavirus, COVID-19, non-invasive vagus nerve stimulation, taVNS, tDCS, TMS, neuromodulation, NIBS

## Abstract

**Background:** Novel coronavirus disease (COVID-19) morbidity is not restricted to the respiratory system, but also affects the nervous system. Non-invasive neuromodulation may be useful in the treatment of the disorders associated with COVID-19.

**Objective:** To describe the rationale and empirical basis of the use of non-invasive neuromodulation in the management of patients with COVID-10 and related disorders.

**Methods:** We summarize COVID-19 pathophysiology with emphasis of direct neuroinvasiveness, neuroimmune response and inflammation, autonomic balance and neurological, musculoskeletal and neuropsychiatric sequela. This supports the development of a framework for advancing applications of non-invasive neuromodulation in the management COVID-19 and related disorders.

**Results:** Non-invasive neuromodulation may manage disorders associated with COVID-19 through four pathways: (1) Direct infection mitigation through the stimulation of regions involved in the regulation of systemic anti-inflammatory responses and/or autonomic responses and prevention of neuroinflammation and recovery of respiration; (2) Amelioration of COVID-19 symptoms of musculoskeletal pain and systemic fatigue; (3) Augmenting cognitive and physical rehabilitation following critical illness; and (4) Treating outbreak-related mental distress including neurological and psychiatric disorders exacerbated by surrounding psychosocial stressors related to COVID-19. The selection of the appropriate techniques will depend on the identified target treatment pathway.

**Conclusion:** COVID-19 infection results in a myriad of acute and chronic symptoms, both directly associated with respiratory distress (e.g., rehabilitation) or of yet-to-be-determined etiology (e.g., fatigue). Non-invasive neuromodulation is a toolbox of techniques that based on targeted pathways and empirical evidence (largely in non-COVID-19 patients) can be investigated in the management of patients with COVID-19.

## Introduction

The first cases of novel coronavirus disease (COVID-19) were reported in Wuhan, China, in December 2019 (1). The disease caused by the new severe acute respiratory syndrome coronavirus 2 (SARS-CoV-2) spread rapidly worldwide and affected more than three million people, and killed more than 750 thousand up to July 2020 (1). The virus spreads by droplet transmission and via direct contact with people while they are infectious in both the pre-symptomatic and symptomatic phases, although a potential transmission *via* fecal, urine, aerosol, and fomite have been reported (2, 3).

COVID-19 presents a variety of clinical symptoms from asymptomatic to severe respiratory dysfunction and death. Key symptoms include fever, anosmia, ageusia, vertigo, nausea, headache, lower limb pain, cough, fatigue, shortness of breath, sore throat, arthralgia, chills, vomiting, and others. In more severe cases, the infection can cause pneumonia, severe acute respiratory syndrome, and kidney failure (4), and on rare occasions, stroke (5, 6), and encephalitis (7–9). Systemic issues such as coagulation disturbances/thrombosis (10, 11) and cytokine storm (12, 13) are also relevant, especially to understand how COVID-19 would be associated with nervous system pathology. Risk factors to severe complications are age (more than 65 years old), and comorbidities, such as systemic arterial hypertension, chronic obstructive pulmonary disease, cardiopathies, morbid obesity, diabetes mellitus, and cancer (14, 15). COVID-19 may not only be restricted to the respiratory system but would possibly affect the peripheral (PNS) and central (CNS) nervous systems which appear to have an influence on morbidity and mortality (16). However, this topic is still a matter of debate.

SARS-CoV was detected in the cerebral cortex and hypothalamus of six out of eight confirmed patients, but not in unconfirmed or control patients (17). The virus may invade the CNS via olfactory nerves, and from the guts via the vagus nerve, reaching brainstem nuclei associated with cardio-respiratory control (18), and thalamus, causing autonomic dysfunction and/or neurogenic respiratory failure (19). Inflammatory waves and particles may reach in the supraspinal nuclei in the brainstem and trigger “the inflammatory reflex,” a pathway that has both immunosensing and immunosuppressive functions (20). Thus, the neuroinvasive potential of the SARS-CoV-2 could be related to the severity of some cases (21, 22), and also extend the impact of the disease on cognitive and behavioral aspects. While a growing body of evidence suggests that COVID-19 is associated with neurological diseases (2–4, 23), the potential neuroinvasiveness of the virus and its relation to COVID-19 pathophysiology continues to be deliberated. There are few documented cases of encephalitis (24). It is not clear if CNS pathological findings are a consequence of direct virus infection or consequent to hypoxia (25), and the controversy of SARS-CoV-2 neuroinvasiveness is not resolved

Although it is not clear if COVID-19 affects the nervous system directly, and how this would impact the severity of some cases, the inflammatory nature of the disease is well-recognized (26–29). Despite the uncertainty of the direct involvement of nervous system pathology in the pathophysiology of COVID-19, it is clear that patients present other necessities such as respiratory care and rehabilitation (22, 30–32) and the management of fatigue, and pain (33, 34), for instance. Strategies to control inflammation usually include pharmacological approaches (35–37), but especially given incomplete efficacy and complications in many patients, alternative treatments approaches are relevant. Non-invasive brain stimulation (38–40) and vagus nerve stimulation (41) have the potential to reduce inflammation. These techniques can be used in the management of psychiatric symptoms associated with the COVID-19 pandemic (39, 42, 43). Non-invasive neuromodulation has also shown to be a potent resource in cognitive and physical rehabilitation (44, 45) and could serve additional goals in the management of COVID-19 patients (30).

Here, we review aspects of the SARS-CoV-2 pathophysiology and its relation to the immune response, autonomic balance, neurological, musculoskeletal and respiratory symptoms, and neuropsychiatric aspects of COVID-19. We highlight the potential applications of non-invasive neuromodulation techniques such as transcranial direct current stimulation (tDCS), repetitive transcranial magnetic stimulation (rTMS), and vagus nerve stimulation (VNS) in the treatment of patients with disorders related to COVID-19. We link specific non-invasive neuromodulation techniques to the management of targeted disease aspects.

Non-invasive neuromodulation may manage disorders associated with COVID-19 through four pathways:

1. Direct infection mitigation through the stimulation of regions involved in the regulation of systemic anti-inflammatory responses and/or autonomic responses and prevention of neuroinflammation and recovery of respiration;
2. Amelioration of COVID-19 symptoms of musculoskeletal pain and systemic fatigue;
3. Augmenting cognitive and physical rehabilitation following critical illness; and
4. Treatment of outbreak-related mental distress including neurological and psychiatric disorders exacerbated by surrounding psychosocial stressors related to COVID-19.

The above pathways may be linked. For example, systemic inflammation can occur alongside brain inflammation and fatigue and/or pain, which will all indirectly aggravate psychiatric symptoms (e.g., isolation provoked anxiety). These pathways both in the context of COVID-19 etiology and specific non-invasive neuromodulation therapeutic targets are addressed here, alongside practical considerations for NiN deployment.

## Materials and Methods

This targeted view (5) was steered by groups of authors involved with research in the fields of inflammation and immune responses to infections, autonomic nervous system activity, neurology, psychiatry, psychology, physiotherapy, rheumatology, neuroscience, bioengineering, and non-invasive neuromodulation. All the groups reviewed the literature using relevant keywords in their specific areas, in search for relevant texts, mainly peer-reviewed articles, to describe a rationale on the use of NiN in the treatment of patients with disorders related to COVID-19. The key problems to be addressed were described by clinicians in reference hospitals in Brazil dealing with COVID-19 patients, and were summarized as: (a) how to help patients who arrive at hospitals with high levels of inflammatory markers, many of which are sent after a short time to intensive care units, and some die after a few days? (b) how to help weaning from mechanical ventilation, intra-hospital rehabilitation, and discharge of patients with COVID-19, who seem to present a slower pattern of recovery, compared to patients without COVID-19? (c) how to approach patients and health teams who are presenting elevated levels of distress, including outbreaks of anxiety; (d) how to prepare for the post-COVID-19 phase, where some patients will need to be rehabilitated because of the consequences of the infection?

After searching the peer-reviewed and pre-print literature and summarizing their findings, key authors from each group joined to integrate their findings, aiming to describe which pathophysiological mechanisms would be approached by the use of NiN. Finally, three authors (BWB, JAC, and MB) externally reviewed the manuscript. The non-invasive neuromodulation tools found to be of relevance were tDCS, rTMS, and VNS. The basis for its use and practical aspects of the application in patients with COVID-19 are described.

### Rationale for the Use of Non-invasive Neuromodulation Techniques in the Treatment of COVID-19 Patients

This section presents the theoretical basis that would underpin the use of non-invasive neuromodulation techniques in the management of COVID-19 patients. The potential neuroinvasiveness of COVID-19 represents the first avenue where these nervous system stimulation techniques would act in the control of the disease. In addition, non-invasive neuromodulation can also stimulate the neuroimmune response to the virus, a key factor to determine the severity of the symptoms. Non-invasive neuromodulation techniques may also be useful in the physical and cognitive rehabilitation of the patients, as well as in the management of the mental health both in patients and healthcare teams.

#### Potential Neuroinvasiveness of COVID-19

**Figure 1:**
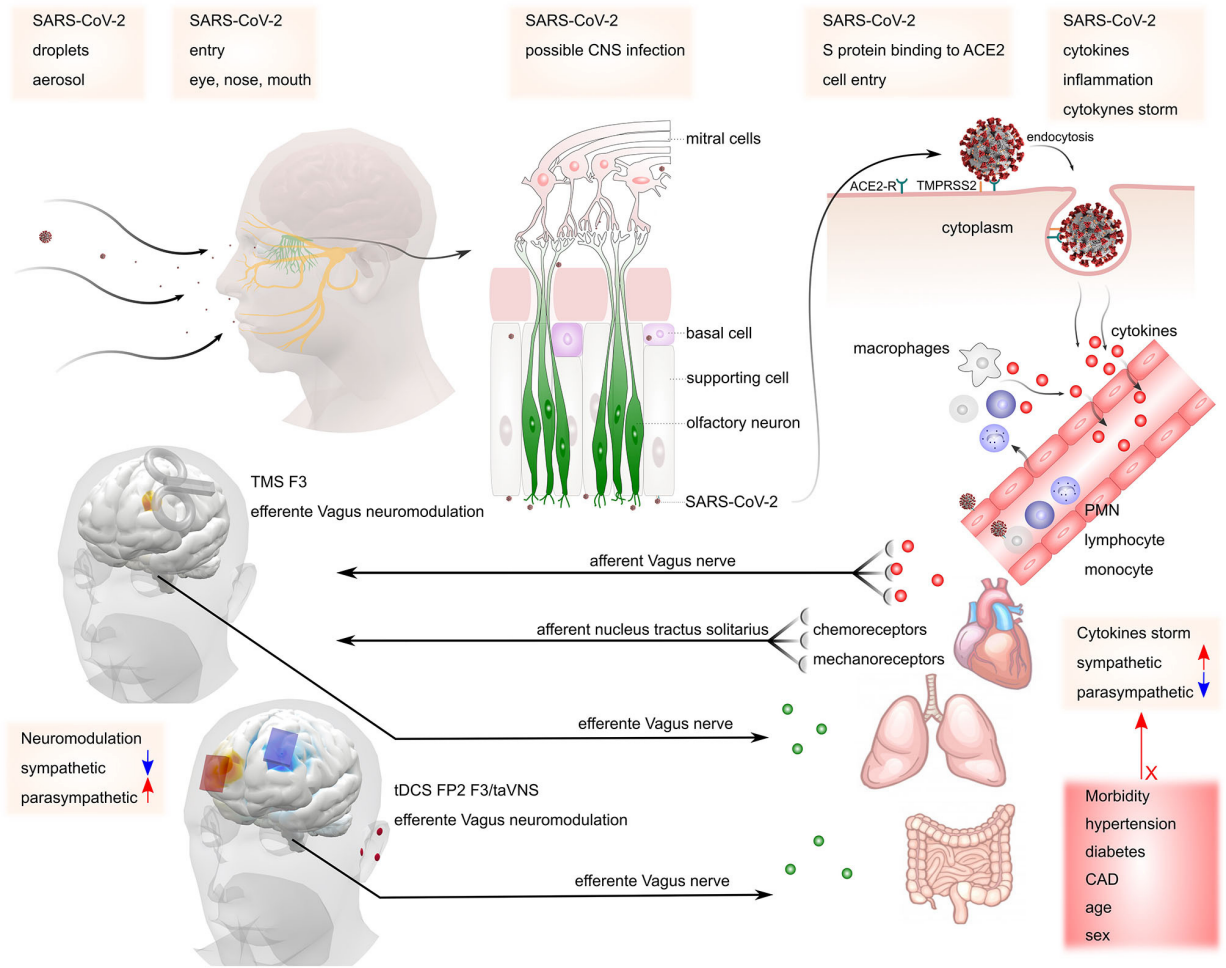
Possible mechanisms of SARS-CoV-2 invasion in the nervous system. SARS-CoV-2 may gain access to the central nervous system via peripheral nerves such as olfactory and vagus nerves. The virus binds to ACE2 receptors, starting the release of cytokines (cytokine storm). This process increases sympathetic activity, which may be responsible for maintaining the inflammatory condition. The presence of co-morbidities such as hypertension, diabetes, coronary artery disease (CAD), increased age, and male sex may contribute to the increased risk of complications. Stimulation of parasympathetic activity *via* TMS or tDCS at the left dorsolateral prefrontal cortex (F3) or transcutaneous vagus nerve stimulation at the ear may counteract increased sympathetic activity mediated inflammation.

SARS-CoV-2, such as MERS-CoV and SARS-CoV can be transmitted through infectious droplets, via angiotensin-2 converting enzyme (ACE2) and transmembrane serine protease 2 (TMPRSS2), which are important to cell viral invasion (46–48). SARS-CoV-2 can directly access the central nervous system (CNS) through the circulation or cranial nerves and the olfactory bulb (18, 49), by synapse-connection (Figure 1) (50–52). In addition, direct endocytotic infection (similar to that demonstrated for the ZIKA and TBEV viruses) may also be a pathway for CNS invasion. Once within the CNS, coronaviruses affect astrocytes, neuroblasts, and neurons (53–55). The neurobiological mechanism involves a direct binding of SARS-CoV-2 to the ACE2 receptor leading to a fall in ACE2, which is responsible for mediating neuroinflammation, neurodegeneration, and neurotoxicity processes related to CNS disorders. Invasion of the brainstem may be also clinically relevant, since the nucleus of the solitary tract (NTS) and nucleus ambiguous are crucial for the maintenance of cardiorespiratory homeostasis (22, 51). Afferents of the vagus nerve convey peripheral inflammation information to the CNS, specifically in the medullary NTS and nucleus ambiguous (56, 57). The NTS responds to hypoxia and hypercapnia by activating or inhibiting the sympathetic activity (58–61). This autonomic response is a powerful regulator of the innate and adaptive immune system (62, 63).

The sympathetic nervous system promotes pro-inflammatory responses, *via* catecholamine release and beta-adrenergic stimulation, and the parasympathetic nervous system promotes anti-inflammatory effects (64). Besides, primary and secondary immune organs have substantial sympathetic innervation and almost all immune cells express receptors for neurohormones and neurotransmitters (65). These factors suggest that COVID-19 may be a systemic disease associated with systemic inflammation and trigger a massive neuroinflammatory response, manifested by reactive astrogliosis and microglial activation (66).

Although respiratory (nasal/oral cavity, pharynx, larynx) to nervous system transmission is still under investigation in the case of COVID-19 pandemic, the reports of neurological symptoms in infected patients support the potential neuroinvasiveness of SARS-CoV-2. Patients with COVID-19 in hospitals of Wuhan presented acute CNS symptoms, such as dizziness, headache, impaired consciousness, acute cerebrovascular disease, ataxia, and convulsions (67). Earlier studies also reported the presence of SARS-CoV within the brain of infected individuals (17, 68), and in the brainstem of animal models (17, 68, 69), supporting the evidence that COVID-19 affects the CNS (22), and also a possible bidirectional communication with the immune system (63). Moreover, other short-term neurological symptoms observed in COVID-19 patients could also be a manifestation of CNS invasion, such as high-grade fever, hypoxia, respiratory, and metabolic acidosis at an advanced stage of the disease (16).

However, a recent study reported inconsistent results on SARS-CoV-2 invasion of the CNS, and one cannot rule out that all the above-described neurological symptoms could be secondary to a non-neurological process (e.g., general inflammation, cytokine storm, hemodynamic shock, systemic thrombotic phenomena). Characterizing the symptoms and etiology of COVID-19 neurological manifestation is complex and subject to ongoing studies, including post-pandemic consequences of the infection, such as encephalitis (70, 71), acute flaccid paralysis (72), acute disseminated encephalomyelitis (19, 73, 74), neuropsychiatric and cognitive impairments consequent of neuroinflammation together with prolonged hypoxia (18). A recent report suggests that neurological manifestations in COVID-19 patients should be classified as confirmed, probable or possible/suspected based in a WHO classification (6). This effort will probably be useful to shed light into the clarification of the involvement of the nervous system pathology into COVID-19. Non-invasive neuromodulation techniques are currently used and trialed for the management of a broad range neurological diseases (7, 8), and are thus candidates in the management of neurological manifestations of COVID-19—as considered later in this article.

#### Immune Response to COVID-19

The innate immune system can recognize lipopolysaccharides, viral antigens, and viral genomes through pattern recognition receptors (PRRs), leading to the activation of intrinsic signaling pathways and the production of pro-inflammatory and anti-inflammatory cytokines (75–78). An immune response initiates after a virus invading the body (host) is recognized by the host innate immune system through PRRs (79, 80). The expression of inflammatory factors, maturation of dendritic cells, and synthesis of type I interferons (IFNs) are induced by the virus, limiting the spreading of the virus while stimulating macrophages (81).

Notwithstanding this innate immune response to the virus, Lu et al. (82) argued that the N protein of SARS-CoV could aid the virus in “escaping” from the expected immune responses. After the initial activation of the innate immunity, the adaptive immune response is involved in a battle against the virus (82). The T lymphocytes (T-cells; CD3+, CD4+, and CD8+) and B-cells (CD19+, CD20+, CD22+), the cellular adaptive components, play an important and complex role in the body defense. For example, CD4+ T-cells stimulate B-cells to produce virus-specific antibodies, and CD8+ T-cells have the function to kill the virus-infected cells. Moreover, the helper cells will produce pro-inflammatory cytokines to aid in the defense. Indeed, humoral immunity is also essential in fighting against the virus in order to combat the viral infection (82, 83). However, SARS-CoV-2 can inhibit T cell functions by inducing apoptosis of T-cells, and an overreaction of the immune system could exaggerate elevating the number of free radicals locally that in turn could lead to damages to the lungs, organs, and. to multi-organ failure and even death (84).

A cytokine storm results from an overreaction of the immune system in SARS and MERS patients (84–86), which releases excessively free radicals and causes acute respiratory distress syndrome and multiple organ failure (87). Therefore, a cytokine storm is a systemic inflammatory response due to a release of cytokines such as TNFα, IL-1β, IL-2, IL-6, IFNα, IFNβ, IFNγ, and MCP-1 (80), and activated macrophages responsible for pro-inflammatory mediators such as cyclooxygenase and nuclear factor-kappa B (88). Sustained inflammatory responses may be related to the critical conditions of COVID-19 patients, whereas those patients admitted in the intensive care unit had higher plasma levels of TNFα, IL-2, IL-7, IL-10, GSCF, IP10, MCP-1, and MIP1A, indicating that the cytokine storm is related to disease severity (84, 85, 89).

Therapeutic immunosuppression is fundamental and critical in the treatment of cytokine storms, notably, in COVID-19 severe conditions. Mehta et al. (12) reported a subgroup of patients with severe COVID-19 that might have cytokine storm syndrome. Huang et al. described patients with COVID-19 in Wuhan (China), presenting high amounts of IL-1β, IFNγ, IP10, and MCP-1, probably leading to activated Th1 cell responses (12, 89). Huang et al. (89) also described that SARS-CoV-2 induces an increased secretion of Th2 cytokines (e.g., IL-4 and IL-10) that suppress inflammation, differently to those observed from SARS-CoV infection. These mechanisms may also be related to the genesis of acute cerebrovascular disease and acute hemorrhagic necrotizing encephalopathy (90), resulting from blood-brain-barrier damage (91). Indeed, data from mouse models suggest that the influenza virus can aggravate ischemic brain injury by triggering a cytokine cascade (92). As all the above mentioned immune responses are linked to peripheral nervous system (PNS) and CNS activity through autonomic responses, nervous system activity may be a key factor in the response to infection, which could in turn be modulated by non-invasive neuromodulation techniques especially through vagus nerve stimulation.

#### Autonomic Response in COVID-19 Infection

The vagus nerve releases acetylcholine (ACh) in the periphery to activate parasympathetic responses in target organs throughout the body such as lowering heart rate HR) and myocardial contractility in the heart (93). There are numerous downstream effects of ACh release in the periphery, such as activating α7 nicotinic ACh receptors (α7nAChR) on macrophages (94–99), inhibiting the production of IL-1, IL-6, IL-18, and HMBG1 (100–102) in several tissues and organs, such as the spleen, intestine, liver, heart, and lung (20, 103). The α7nAChR has an important role in the control of inflammation since α7nAChR-deficient mice show higher levels of pro-inflammatory cytokines in blood, spleen, and liver after endotoxin when compared to wild-type mice (104). In addition to that, ACh is also released by T and B cells with autocrine responses such as IL-2 release and T cell proliferation (105, 106), corroborating its importance in the inflammatory modulation.

Vagal activity is correlated with decreased inflammatory markers (e.g., IL-6, C-reactive protein) (107, 108). In experimental models, lesioning the vagus nerve (vagotomy) exacerbates the inflammatory response in colitis, pancreatitis, viral myocarditis, and sepsis (109–111). It also increases the synthesis of pro-inflammatory lipid mediators, while decreasing pro-resolving mediators such as netrin-1 and specialized pro-resolving mediators (SPMs) (112), which decrease the resolution of bacteria inflammation (113, 114). In addition, vagotomy not only decreases ACh release but also catecholamines (113, 114), which likewise have an important role in controlling inflammation (115). Deficiency in T- and B-cells related to increasing in alternatively activated immune cells lead to exacerbated viral replication, prolonged inflammatory responses both systemic and locally, induction of procoagulant factors, hemodynamic changes, ischemia and thrombosis leading to poor outcomes (116–118). The presence of SARS-CoV-2 in the brainstem, independently of the infection detected in the lungs, induces neuronal loss and dysfunction (119), which may be associated with an autonomic imbalance with a decrease of ACh and catecholamine release in the periphery. It is noteworthy that cardiovascular disease and diabetes, risk factors for worse prognostic and death by the COVID-19 (120–122), are characterized by decreased autonomic function. This condition may be relevant in some COVID-19 patients who present a high inflammatory profile and could be targeted by strategies to increase vagus activity, which have already been shown to regulate autonomic function in patients with cardiovascular diseases and diabetes.

#### Musculoskeletal Symptoms and Fatigue in COVID-19

Musculoskeletal symptoms and fatigue in COVID-19 may also represent the affection of nervous and/or immune function. Skeletal muscle symptoms were shown to be common in individuals with COVID-19 (67). Different meta-analyses of COVID-19 clinical characteristics have reported an incidence of generalized myalgia and/or fatigue that ranges from 35.5 to 42.5% (33, 34, 123). Muscular symptoms are the third or fourth more frequent manifestation reported by individuals. Also, these symptoms should be taken into account for diagnostic criteria, since individuals with severe infection are more likely to present non-typical symptoms first (67). Individuals with muscular symptoms presented an increased inflammatory response, including higher levels of C-reactive protein (67). These findings are indicative of muscle injury, although the lysis of striated muscle is considered as a rare complication of COVID-19 (34).

There are two proposed mechanisms to explain myalgia and fatigue. The first is that the inflammatory response is not only the consequence of muscle injury but also the cause. Not only individuals with a more severe infection have more incidence of muscle symptoms, but also those who present muscle symptoms usually have multiple organ lesions (67). Altogether, there is some evidence that systemic inflammation can lead to muscle fiber necrosis (124). The second mechanism is related to the ACE2 receptor targeted by the virus and also found in muscle cells (67, 124). It is hypothesized that COVID-19 could injure directly the muscle tissue, but there is no evidence to substantiate this theory, and it comes from studies of SARS. Two studies conducted an analysis of post mortem muscle tissue of patients who died with SARS (68, 125). One of them did not find any evidence of the virus in muscle tissue (68). The other found focal myofiber necrosis but with small quantities of inflammatory infiltration (125). Authors of the second study argue about not being able to remove the confounding influence of mechanical ventilation used by these patients, and its side effects on their findings. Probably, the systemic inflammatory response is the main cause of muscle symptoms in individuals with COVID-19. Muscle fatigue and weakness could hamper respiratory function and become a vicious cycle with the aid of mechanical ventilation devices, which *per se*, can cause more weakness (125).

#### Psychiatric Symptoms and the Mental Health Outbreak Related to COVID-19

The evidence of the impact of this pandemic on mental health is evolving. An online survey of 714 Chinese patients with stable COVID-19 disease reported a 96.2% prevalence of significant post-traumatic stress symptoms (126). As for the general population, a survey of residents of Wuhan and surrounding cities, the epicenter of the China outbreak, the prevalence of post-traumatic stress symptoms was 7% as assessed up to 2 weeks after mandatory quarantine for all citizens was implemented. Women and those with sleep complaints were reported to be at increased risk (127). As for protective factors of anxiety symptoms, family income stability, and living with parents were protective (128). Among healthcare professionals, 28% of nurses and physicians working in Wuhan were found to have either moderate or severe symptoms in the domains of depression, anxiety, insomnia, and distress (129).

### Potential Use of Non-invasive Neuromodulation on COVID-19 Related Disorders

In the previous section we described how COVID-19 may affect or be mediated by the nervous system and immune activity, aspects that can be targeted by non-invasive neuromodulation techniques in order to manage the disease. We now present the rationale specific uses of these techniques in the management of COVID-19 patients, relying largely on evidence from relevant non-COVID-19 populations, as direct trials of non-invasive neuromodulation in COVID-19 patients remain limited or are ongoing.

**Figure 2:**
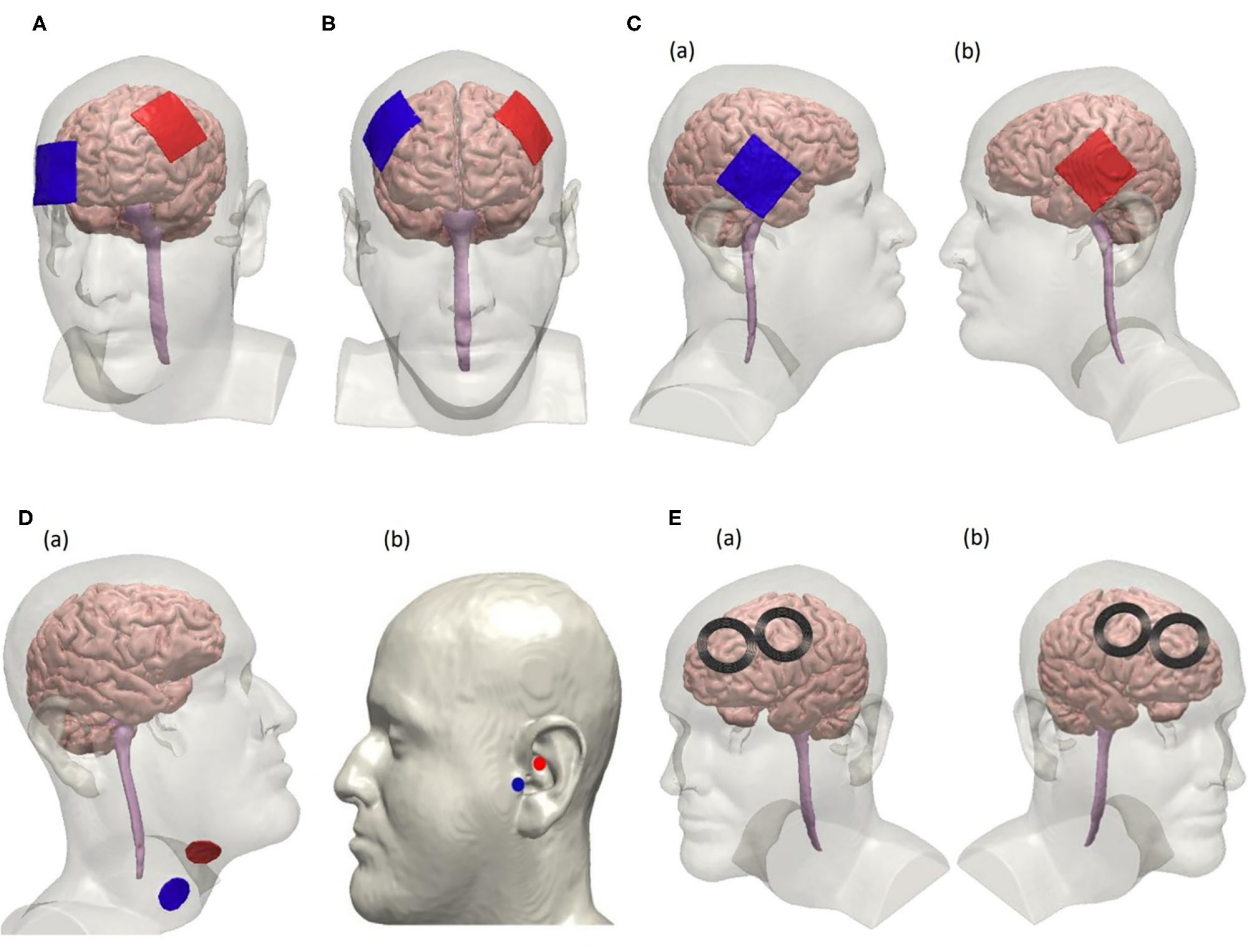
Electrode configurations for non-invasive tDCS, VNS, and rTMS following the 10–20 EEG system. **(A)** Unilateral tDCS with anode positioned over F3 and cathode over Fp2 on the scalp to modulate the left dorsolateral prefrontal cortex (DLPFC). **(B)** tDCS using a bifrontal montage to perform anodal stimulation on left DLPFC where the anode is positioned over F3 and cathode is positioned over F4. **(C)** Anodal tDCS to stimulate the temporal cortex using a bifrontal configuration where the cathode is positioned over T4 and the anode over T3 as seen in (a,b), respectively. **(D)** Non-invasive vagus nerve stimulation by modulating the cervical branch of the vagus nerve in (a) and the ear in (b). Electrode placement for cervical vagus nerve stimulation is shown in (a). Electrodes are placed at the tragus and the cymba conchae of the left ear to perform unilateral taVNS as shown in (b). **(E)** rTMS using a figure-8 coil positioned over F3 to stimulate the left DLPFC suggested for high-frequency protocol is shown in (a). Right DLPFC is stimulated using the low-frequency rTMS protocol by placing the coils over F4 as shown in (b).

The possible presence of an autonomic imbalance in COVID-19 and the importance of vagus nerve activity in the control of inflammation may represent key features to the use of NiN in the treatment of COVID-19 patients, markedly those with high levels of inflammatory profile. Vagus nerve activity can be increased via the cerebral cortex through areas that modulate it indirectly such as the left dorsolateral prefrontal cortex (DLPFC) (130), corresponding to the F3 position of the 10–20 International EEG System, or temporal cortices. Also, the vagus nerve innervates the ear, mainly the pinna of the outer ear (131), making it possible to stimulate these areas transcutaneously to influence vagus activity (9, 10). In this section, we will review the most promising, readily available NiN approaches that modulate the central and peripheral immune response. At the same, NiN may be useful in the control of musculoskeletal psychiatric symptoms and through the same or even different cortical targets as those used in the control of inflammation. The subsequent sections will present the basis for the use of NiN in the treatment of COVID-19 patients using techniques such as rTMS, tDCS, and vagus nerve stimulation directed to the DLPFC, motor cortex, and where the vagus nerve is superficially accessible (Figure 2).

#### Left Dorsolateral Prefrontal Cortex Neuroimmune Modulation With TMS or tDCS

The vagus nerve is responsible for the parasympathetic innervation of the heart; its stimulation decreases heart rate (HR), and interferes with heart rate variability (HRV) (132, 133). This phenomenon was also observed when stimulating other areas of the CNS, such as the DLPFC (134), perigenual, and mid-anterior cingulate cortex (pgCC and maCC) (135, 136), which lead researchers to suggest that those cortical areas modulate vagal activity.

Studies with transcranial magnetic stimulation (TMS) have helped elucidate the relationship between the DLPFC and vagus nerve activity. Iseger et al. (134) applied trains of high-frequency 10 Hz rTMS over 10 cortical regions aiming to identify which regions would affect HR. They found that 20–40% of the participants presented decreased HR and heart rate variability (HRV) with stimulation of the DLPFC, either left (F3, FC3; 10–20 EEG System) or right (F4, FC4; 10–20 EEG System). Interestingly, stimulation of the motor (C3, C4–10-20 EEG System) and parietal (Pz–10–20 EEG System) cortices showed opposite effects. Effects were more pronounced in the right DLPFC, which is contrary to other studies showing that stimulation of the left, but not the right DLPFC changes HRV (137). The variability found in these studies is probably because of individual patterns of connectivity between the DLPFC and other cortical and subcortical structures. As an example, in a TMS/fMRI, Iseger et al. (134) and Vink et al. (138) found that only four under 10 participants had the subgenual cingulate cortex activated by stimulation of the DLPFC.

The anti-inflammatory effects of DLPFC stimulation support the idea of DLPFC/vagus connectivity. Aftanas et al. (139) applied dual-target rTMS to the motor cortex (bilaterally; 10 Hz; 100% of resting motor threshold; 4,000 pulses) and to the left prefrontal cortex (DLPFC; 10 Hz, 110% of resting motor threshold; 3,000 pulses) for 20 days in patients with Parkinson disease. They reported significant down-regulation of the spontaneous production of pro-inflammatory cytokines. Although not tested in this study, the effect may be related to increased vagal activity and suggests that the DLPFC would be a potential target in the control of inflammatory cascade in patients with COVID-19.

Transcranial direct current stimulation (tDCS) has also been used to probe HRV and vagal activity. Carvenali et al. stimulated the left DLPFC with anodal tDCS immediately before and during exposure to stress and showed decreased HRV only in the period prior to stress exposure (140). Similar results were found with bifrontal tDCS (141), which raises again the question about the effect of laterality when stimulating the DLPFC with the aim to increase vagus nerve activity.

Taking together, those studies suggest that stimulation of the DLPFC with rTMS or tDCS could be useful to increase vagal activity. The consequent decrease in inflammation with those strategies is speculative. However, as patients with COVID-19 also present mood disturbances such as stress and anxiety, targeting the DLPFC would be useful in the control of inflammation and neuropsychiatric problems associated with the infection (see below for neuropsychiatric effects). Unilateral tDCS protocols would target the left DLPFC with the anode positioned over the F3 scalp position (10–20 EEG System), and cathode over the Fp2 scalp position (10–20 EEG System) or another distant location. Typical current intensities of 1–2 mA, for 20–30 min, and electrodes' sizes ranging from 5 × 5 cm or 7 × 5 cm. Bifrontal protocols would position the anode over F2, and cathode over the F4 scalp position (10–20 EEG System) or in the bifrontal “OLE” montage which can optimize current delivery to DLPFC (11–13). Targeted tDCS of DLPFC can be achieved using 4x1-tDCS centered over DLPFC (142, 143). High-frequency rTMS protocols would target the left DLPFC with 10 Hz, trains of 50 pulses, with intertrain intervals of 25–50 s, at 90–120% of the resting motor threshold, until 3,000 pulses per session, with figure-of-eight coils positioned at the F3 scalp position or using neuronavigation (14). The right DLPFC may be targeted with low-frequency 1 Hz rTMS, maintaining the same intensity and number of pulses of high-frequency rTMS. More details for rTMS treatment can be found in the study of Pereira et al. (144).

#### Temporal Cortex Autonomic Modulation With tDCS

The use of tDCS over the temporal cortex aims to reach the insular cortex, an area beneath the temporal cortex with profuse autonomic and limbic connectivity. Intraoperative electrical stimulation of the left insular cortex increased blood pressure and HR and stimulation of the right insular cortex resulted in opposite effects (145). In addition, left insular cortex lesion resulted in perturbations of the cardiac autonomic function in humans (increased cardiac sympathetic tone and decreased parasympathetic tone) and predisposed individuals toward a pro-arrhythmic state (145). Furthermore, neuroimaging studies have shown the relation between the insular cortex and cardiac autonomic control (146, 147), and acute ischemia of the insular cortex was independently associated with poststroke hyperglycemia, which may reflect sympathoadrenal dysregulation, although no evidence of lateralization was found (148). Other studies have suggested a role played by the insular cortex in a phenomenon called post-exercise hypotension (e.g., temporary decrease in blood pressure below pre-exercise values) (149, 150). Hence, the temporal cortex has been the target in several studies aiming to modulate cardiac autonomic control or other functions associated with the insular cortex (151–155).

Montenegro et al. (155) assessed the effects of anodal tDCS (2 mA for 20 min) over the left temporal cortex on measures of cardiac autonomic control at rest in two groups of healthy adults, a group of athletes and a group of non-athletes. The stimulation improved cardiac autonomic control in athletes but not in untrained individuals, namely parasympathetic activity, increased whereas the sympathetic activity decreased. The authors attributed the specific results to neuroanatomical and functional changes in the brain induced by long-term exercise training (155). Furthermore, Piccirillo et al. (154) demonstrated that anodal tDCS over T3 scalp position (10–20 International EEG System), 2 mA for 15 min, improved temporal ventricular repolarization dispersion, reduced sinus sympathetic activity and systemic peripheral resistance, and increased vagal sinus activity and baroreflex sensitivity in older (>60 years old; mean age 70 ± 6 years), but not younger (<60 years old; mean age 36 ± 11 years) individuals. It should be noted that older individuals are at increased risk for worse prognosis and death (120, 121, 156).

Interestingly, besides modulating cardiac autonomic control at rest, tDCS over the left temporal cortex may also modulate autonomic control during exercise (151, 152). Okano et al. (152) applied anodal tDCS over T3 scalp position (2 mA for 20 min) in a sample of elite cyclists before submitting them to a maximal graded cycling exercise test (e.g., stress test) and found that the stimulation decreased heart rate at submaximal intensities for roughly half of the exercise test duration. These results were also replicated by Kamali et al. (151) who found decreased HR during fatiguing knee extension exercise after concomitant anodal tDCS over T3 and primary motor cortex (M1) (2 mA for 13 min) in trained bodybuilders.

Taken together, these results demonstrate that anodal tDCS applied over the temporal cortex may improve autonomic function in healthy individuals at rest and during stressful stimuli (e.g., exercise). Respiratory exercises are being used in patients with COVID-19, and temporal cortex tDCS may be used to increase their effectiveness in restoring respiratory function in these patients. It is suggested that tDCS may be used with the anode positioned at the T3 scalp position, and cathode at the T4 scalp position, with 2 mA for 20 min, using 5 × 5 cm or 5 × 7 cm electrodes, together with exercises directed to respiratory function. For targeted temporal cortex tDCS the 4x1 HD-tDCS can be used (142, 143).

#### Neuroimmune Modulation Through Transcutaneous Vagus Nerve Stimulation

The vagus nerve plays a central role in the autonomic nervous system. It mediates major visceral functions such as heart rate, gastrointestinal motility and secretion, pancreatic endocrine and exocrine secretion, hepatic glucose production, and other visceral functions. Furthermore, and most relevant to the current pandemic is that activation of the vagus nerve suppresses immune and inflammatory responses to pathogen invasion and tissue injury (157). Modulating the vagus nerve has been demonstrated to suppress inflammation is being explored as a treatment for pulmonary arterial hypertension and COPD-related bronchoconstriction (ClinicalTrials.gov:NCT01612793).

The vagus nerve may be stimulated invasively and non-invasively. Cervically-implanted vagus nerve stimulation (VNS) activates the parasympathetic system and mediates lymphocytes and macrophages inhibiting pro-inflammatory production (113, 158–160), improving survival in experimental sepsis, hemorrhagic shock, ischemia-reperfusion injury, and other conditions of cytokine excess (161). Interestingly, VNS increases dopamine levels, and similarly to ACh, dopamine also shows anti-inflammatory mechanisms by decreasing TNF-α and inflammasome after endotoxins (113, 114). VNS not only inhibits the inflammation but also induces the expression of the SPMs, including the lipoxins, resolvins, protectins, and maresins (162). VNS also regulates the SPMs expression, polymorphonuclear infiltration, and the chemokines and cytokines release, which are directly involved in the inflammatory inhibition within the nervous system (162). Hence, the so-called pro-resolution vagal reflex (163), may induce a more efficient resolution of the inflammatory storm in COVID-19 patients, helping also to improve the quality of life and survival expectancy in these patients.

Recently, a non-invasive form of VNS known as transcutaneous VNS (taVNS) has emerged as a promising, non-invasive alternative to its surgically-implanted predecessor. taVNS activates the vagal system by delivering electrical pulses to the auricular branch of the vagus nerve (ABVN) that innervates both left and right ears (164). taVNS is simple and inexpensive to administer, requiring only bipolar electrodes attached to the skin mainly in the tragus and cymba conchae (131, 165). A consensus on optimal stimulation parameters is yet to be determined, however, taVNS is generally administered using the following range of waveforms: monophasic or biphasic pulses delivered at 5–25 Hz pulsed, ≤ 500 μS pulse width, ≤ 10 mA (166). taVNS can be administered either unilaterally (left ear) or bilaterally (left and right ears) at either the left tragus or cymba conchae, for 1 h sessions (166). The safety and tolerability of this method were assessed in several studies which showed minimal side effects (165, 167–170). It is important however to consider parasympathetic activation *via* taVNS and monitor for cardiac effects (171).

Transcutaneous cervical VNS (tcVNS) is another form of non-invasive VNS that delivers electrical stimulation to the cervical vagus nerve transcutaneously through the neck. Electrodes are placed over the carotid sheath and stimulation is applied with devices that activate the underlying nerve and tissue. tcVNS frequencies range from 5 Hz to 5 KHz (172, 173). A recent paper has proposed, based on two case studies, the use of tcVNS to manage respiratory symptoms in COVID-19 patients (174). They showed that tcVNS decreased the use of opioids and cough suppressant medication, and promoted relief from chest tightness and shortness of breath, improving lung clearance. As both taVNS and tcVNS are very easy to administer and studies have shown they can increase vagus nerve activity, they both are suggested as potential techniques in the treatment of COVID-19 patients to control inflammation and decreased respiratory discomfort associated with respiratory symptoms.

#### Non-invasive Neuromodulation to Target Musculoskeletal Symptoms, Restore Normal Respiration, and Function and to Accelerate Patient Discharge

Musculoskeletal symptoms in COVID-19 are probably a consequence of systemic inflammation, but a key factor to be addressed, as the musculoskeletal system is strongly related to the capacity to move and perform daily life activities, and probably should be addressed early in the treatment of infected patients. Non-invasive neuromodulation techniques could not only reduce the muscle symptoms present in this population but also improve respiratory muscle function (175–178), training (44, 179–181), and fatigue (182), increasing their motivation, and likely positively affecting the cognitive process, which could aid them in the recovery from the illness.

Respiratory dysfunction is a major concern in COVID-19 (183), with many patients submitted to oxygen support and mechanical ventilation (184, 185). It is already known that respiratory dysfunction has a neural correlate which has in part to do with the potential role of the supplementary motor cortex (SMA) in the control of the diaphragm muscle, what has been recently evidenced by the use of TMS (186) and functional magnetic resonance imaging (187). Conditions such as diaphragm loading, and changes in hypoxia and hypercapnia, change transiently diaphragmatic motor-evoked potentials, but these changes may be the source of difficulties in mechanical ventilation weaning.

At present, only one study investigated the effects of mechanical ventilation on cortical excitability and showed that motor-evoked potentials were depressed; one mechanical ventilation was performed non-invasively *via* nasal mask (188). This result highlights the potential role of mechanical ventilation in depressing CNS excitability and raises the question if failure in weaning from mechanical ventilation has a CNS component. Reports from hospitals struggling with the COVID-19 infection have shown that patients stay ~15–20 days intubated and in mechanical ventilation and that weaning off is slow (189). It is possible that this exquisite pattern may be due to the invasion of the CNS by the virus, as previously shown. Non-invasive neuromodulation techniques such as tDCS or rTMS could be used to help the re-establishment of diaphragmatic drive, but there is still not sufficient evidence to support this use. However, one study has shown that tDCS reduced diaphragmatic motor-evoked potentials (190), which would suggest that stimulation of the motor cortex would not help in mechanical ventilation weaning.

#### Non-invasive Neuromodulation on Outbreak-Related Mental Distress

Infectious disease outbreaks pose many challenges to society. As a consequence of fear, stress, social isolation, reduced income, and other factors, psychiatric symptoms may worsen or emerge in those previously asymptomatic people (191). Patients with prior mental illness and frontline healthcare personnel are at an increased risk of psychiatric symptoms during outbreaks (192). Moreover, the impact on mental health on survivors occurs in the long-term, outlasting the pandemic for months to years (193). Non-invasive neuromodulation strategies have been increasingly used as effective clinical interventions in the treatment of diverse neuropsychiatric disorders (194, 195). Amongst non-invasive neuromodulation techniques, the most established on clinical grounds is rTMS, an intervention already approved by regulatory agencies for the treatment of major depression in many countries, such as the United States, across the European Union, Israel, Australia, and Brazil (196). Furthermore, rTMS may also be an effective treatment for anxiety and trauma-related disorders, as shown in a recent meta-analysis (PMID: 31066227). One of the major barriers of the broad use of rTMS as mental health interventions is the non-portability of devices. As such, patients need to move to health care facilities, which can be located either in small clinics or in hospitals, to have access to this therapy. Usually, the acute treatment is performed in daily sessions five times a week for some weeks, while the maintenance treatment is more spaced out, with fewer weekly, biweekly, or monthly sessions (197). Since a relevant number of patients that receive rTMS comprise risk groups for COVID-19 severe outcomes (e.g., elderly, smokers, chronic cardiopulmonary diseases), there is a need for session frequency reduction or postponement in those with relatively controlled symptoms, which should be addressed in a case-by-case approach. On the other hand, for hospitals with non-invasive neuromodulation services, rTMS treatment could be offered both for stable COVID-19 inpatients and healthcare personnel, assuring proper measures to control viral transmission are implemented. For inpatients with COVID-19 and psychiatric symptoms requiring medical intervention, care must be taken in the prescription of psychotropic drugs if antiviral medications are concomitantly administered, in order to avoid harmful drug-drug interactions (198). Antipsychotics such as risperidone, aripiprazole, and haloperidol (199) and antidepressants such as fluoxetine, paroxetine, sertraline, and duloxetine (200) are metabolized by CYP2D6, the same enzyme that metabolizes chloroquine, a current investigational drug for the management of COVID-19 (201). In this context, the use of NiN would be a safer option.

Standard rTMS protocols for the treatment of psychiatric disorders include high (“excitatory”) and low (“inhibitory”) frequency trains with coil positioned in the scalp usually over either the right or left DLPFC, according to the indication (195, 198, 202). Stimulation of the DLPFC can transynaptically enhance activity in the ventromedial prefrontal cortex, which is hypoactive in trauma-related disorders and possibly related to impaired fear responses, a hallmark of these conditions (203). High-frequency rTMS delivered over the right DLPFC has been deemed efficacious for post-traumatic stress disorder (PTSD) in some clinical trials (195, 198, 202) and would be a reasonable approach to treat patients with trauma-related symptoms. However, in the presence of signs or symptoms that suggest CNS organic compromise, “inhibitory” protocols should be preferred in favor of minimizing the theoretical risk of induced seizures. Low-frequency stimulation of the right DLPFC would be an alternative since one trial that compared both high and low-frequency protocols found no superiority of one intervention over the other in the improvement of PTSD symptoms (204). Furthermore, low-frequency pulses delivered to the right DLPFC may also be effective for the treatment of generalized anxiety disorder (205). Hence, the choice of the ideal strategy should be guided individually after a careful psychiatric assessment. Since the length of hospital stay for recovered COVID-19 patients is around 21 days (156), rTMS treatment might be continued after discharge in some cases. As neither the immunization (206) nor transmitter status (207) after COVID-19 symptom resolution is clear yet, hygienization procedures and personal protective equipment use should be maintained during further rTMS sessions.

As for the treatment hospital staff, a small sham-controlled trial delivered high-frequency rTMS stimulation over the left DLPFC in healthcare workers diagnosed with occupational stress. Sessions were performed three times a week for 4 weeks, with significant improvement of symptoms at follow-up (208). Another expedite strategy would be to administer intermittent theta-burst stimulation (iTBS) over the right DLPFC, an approach effective for PTSD (209). TBS is a particular type of rTMS that can be performed in shorter sessions (maximum of 10 min), minimizing the time that healthcare personnel would need to spend in the NiN sector.

As opposed to rTMS, tDCS could safely be instituted in different environments, including the domestic or ICU setting. However, evidence of tDCS efficacy in anxiety and stress disorders is evolving (194, 210). Bifrontal 2 mA stimulation with cathode over the right and anode over the left DLPFC was performed on a recently published controlled randomized trial, supporting the efficacy of tDCS in the treatment of PTSD (211). In contrast, evidence for tDCS in the treatment of generalized anxiety disorder is less robust (212). A randomized controlled trial showed improvement in physical stress symptoms but not in the primary outcome measure (Hamilton Anxiety Rating Scale) in the active group in comparison to sham (213). Therefore, should tDCS be prescribed, the anode should overlie the left DLPFC with cathode either on the right DLPFC (preferably) or over the right supraorbital area. The current intensity should be set at 2 mA for 20 min, with 5 or 10 (preferably) daily sessions.

#### Home Use of Non-invasive Neuromodulation

Prior to the COVID-19 pandemic, the availability of home-based non-invasive neuromodulation was already compelling and developed (214–218), especially noting the inconvenience for patients already struggling with debilitating disorders to travel daily for brief non-invasive neuromodulation treatment. Home-use tDCS could also offer an advantage in the context of limited outpatient resources for people living in remote areas (219). Home-base treatment is taken on increasing importance with travel and in-patient treatment severely constrained by the COVID-19 pandemic (e.g., many ill patients confined to the domestic environment).

In the context of clinical care, recommendations for home-based non-invasive neuromodulation involve intervention (device, patient) specific levels of oversight to ensure compliance and tolerability. The remote-supervised tDCS rubric provides specific guidance (16), that is directly applied through the COVID-19 pandemic (218). The need for devices that can be reprogrammed remotely, video-conferencing, and accessories to support reproducible stimulation are all intervention specific considerations. For example, online monitoring with a video conference, so that the supervisor can check the correct positioning of electrodes may be deemed important only a first on-boarding session, for only a few sessions until subject competence is confirmed, or every session (214, 216, 220, 221).

Candidates for home use should receive the device from care providers, along with appropriate training on a physical encounter, to minimize the risk of misuse and overuse (194). Training should cover sponge preparation, electrode placement, stimulation initiation with ramp up until the target intensity, and standard operating procedures for troubleshooting common problems. Customized head-bands that facilitate electrode positioning and improve compliance could also be provided (142, 220, 222). Parameters used in most clinical trials are similar to what is usually employed in care facility settings (220). Therefore, tDCS interventions outlined previously could be recommended for home use (216).

#### Practical Aspects and Devices Hygienization

As with all COVID-19 safety procedures, regional and institutional guidance, applied judiciously to specific protocols considering changing conditions, will determine which procedures should be implemented and which should be abbreviated (143). Social/physical distancing parameters as defined by governments and regional regulatory authorities vary and change over time as regional COVID-19 situations evolve. Any recommendations, including the following discussion, is, therefore, region and institute specific, and subject to ongoing risk-burden evaluation. Frequent and adequate hand hygiene is one of the most important measures that should be used to prevent infection by the COVID-19 virus (223). Professionals should perform more frequent and regular hand hygiene, with appropriate techniques (224), including before and after NiN sessions. In addition, the use of personal protective equipment (PPE) is recommended for the provision of health care with direct contact with infected patients, and include gloves, medical masks, goggles or face shields and gowns, and for specific procedures, respirators and aprons (225).

In inpatient units, the use of an apron is recommended for each suspected case. However, considering that overuse of PPE may impact supplies in situations of shortages (225). Following this rationale, in relation to non-invasive neuromodulation, appropriate protocols should be implemented for the single-use or cleaning or components—this applies not only to accessories that both contact patients (e.g., headgear) but to all surfaces, equipment, and cables (e.g., lead wires from the device). Still, additional precautions should be taken in patients under isolation because of COVID-19 and other infections (e.g., acinetobacter, clostridium), to avoid the risk of cross-infection.

Some essential aspects must be pointed out in relation to the hygiene of non-invasive neuromodulation equipment^1^. The recommended cleaning and disinfection procedures for healthcare equipment must be followed consistently and correctly. In particular, for the disinfection of small surfaces between uses (reusable equipment), the use of 70% ethyl alcohol is recommended (223). In cases of application of TMS, it may be important to use support for fixing the coil. If another associated intervention is not necessary, the therapist must maintain a distance of at least one meter, always monitoring the session and the patients' signals. We emphasize, as with any COVID-19 safety protocols, the appropriateness of googles, visors, protective visors, and other PPE, and relevant distancing protocols, for specific social and clinical environments, will ultimately be guided by on the current regional and institutional rules.

## Conclusions

This paper presents empirical evidence, theoretical foundations, and rationale for the potential use of non-invasive neuromodulation techniques such as tDCS, rTMS, taVNS, and tcVNS in the management of COVID-19 related disorders. These techniques may be useful to modulate peripheral and central inflammation response, musculoskeletal and respiratory symptoms, and mental distress associated with COVID-19, even though neuroinvasiveness is still unclear. Thus, the potential benefits of non-invasive neuromodulation should be an important component of ongoing COVID-19 treatment research. Contributing with these international efforts, we made a web-based open-access guideline resource that centralizes all available information related to the use of non-invasive neuromodulation techniques in the management of COVID-19 symptons, including research results and treatment protocols.

## Author Contributions

All authors listed have made a substantial, direct and intellectual contribution to the work, and approved it for publication.

## Conflict of Interest

AFB and KMS are an instructor at the non-invasive neuromodulation course of São Paulo University (USP)/Brazilian Association of Neurofunctional Physiotherapy (ABRAFIN)/NAPeN. BB is listed as an inventor on brain stimulation patents assigned to the Medical University of South Carolina, has equity in Bodhi NeuroTech, Inc. KMS is the coordinator of the non-invasive neuromodulation course of Saulo University (USP)/Brazilian Association of Neurofunctional Physiotherapy (ABRAFIN)/NAPeN. The City University of New York holds patents on brain stimulation with MB as inventor, has equity in Soterix Medical Inc., consults, received grants, assigned inventions, and/or serves on the SAB of Boston Scientific, GlaxoSmithKline, Mecta, Halo Neuroscience, X. The remaining authors declare that the research was conducted in the absence of any commercial or financial relationships that could be construed as a potential conflict of interest.

## Footnotes

^1^https://psyarxiv.com/82bmu/ (accessed May 12, 2020).
